# Whole-Genome Sequence of *Streptococcus suis* Serotype 3 Strain YB51

**DOI:** 10.1128/genomeA.00884-13

**Published:** 2013-10-31

**Authors:** Kaicheng Wang, Jiming Chen, Huochun Yao, Chengping Lu

**Affiliations:** China Animal Health and Epidemiology Center, Qingdao, Chinaa; College of Veterinary Medicine, Nanjing Agricultural University, Nanjing, Chinab

## Abstract

We report here the second complete genome sequence of *Streptococcus suis* serotype 3 (strain YB51). The genome is 2,043,655 bp in length, which is 14,840 bp longer than the first reported genome of the same serotype, and it covers 2,012 coding sequences, 56 tRNAs, and 4 rRNA loci.

## GENOME ANNOUNCEMENT

*Streptococcus suis*, a Gram-positive coccus, can cause a wide range of swine diseases, including meningitis, septicemia, and endocarditis, and it is responsible for severe economic losses to the swine industry worldwide (1, 2). It is also of significance in public health, as humans in close contact with pigs or pork products can be infected with the bacteria through skin wounds or the consumption of raw pork (3, 4). Human infections with *S. suis* commonly lead to meningitis (5). Septic shock, endocarditis, cellulitis, peritonitis, rhabdomyolysis, arthritis, spondylodiscitis, pneumonia, uveitis, and endophthalmitis can also occur (6). During the past several years, the number of *S. suis* infections reported worldwide has increased significantly, with most cases reported in Asia (7).

Currently, 33 serotypes (1, 1/2, 2 to 31, and 33) of *S. suis* have been identified (7). Although there is no clear association between specific serotypes and a given pathological condition, strains isolated from diseased pigs primarily belong to serotype 2 in most countries, followed by serotypes 3, 4, 5, 7, 8, and 1/2 in Asian countries (8–10) and serotypes 3, 1/2, 4, 7, and 8 in Canada (11). Therefore, *S. suis* serotype 3 is rather prevalent worldwide. To facilitate studies and the detection of *S. suis*, we sequenced the genome of a strain of *S. suis* serotype 3. The strain, YB51, was isolated from a clinically healthy pig in China, and its serotype was confirmed using the serotyping antiserum prepared according to previously reported methods (12).

The complete genome sequence was determined by Solexa pyrosequencing at the Beijing Genomics Institute (BGI) (Shenzhen, China). Assembly was performed using SOAP*denovo*. Gaps were filled by primer walking and sequencing of PCR products. The assembly of the genome was further verified by PCR. Coding sequences (CDSs) were predicted using Glimmer 3.02 and GeneMarkS (13) and further examined with the nonredundant protein database through BLASTp. tRNAs and rRNAs were identified using tRNAscan-SE and RNAmmer (14), respectively.

The genome of YB51 was found to be in a similar orientation as the majority of the published genomes of *S. suis* in GenBank (15). The genome of strain YB51 consists of a single circular chromosome that is 2,043,655 bp in length, with a G+C content of 41.28%. The genome is 14,840 bp longer than the genome of the strain *S. suis* ST3 in the same serotype (16). A total of 2,012 CDSs that account for 97.1% of the genome, 56 tRNAs, 4 rRNA loci, and 2 prophage elements were identified in the YB51 genome.

The genome of YB51 harbors some virulence-associated genes, including *srtA, pgdA*, and *fbps* (17). Some frameshift mutations were found in the virulence-associated genes *mrp*, *ofs*, and *zmpC*. Two other virulence-associated genes, *epf* and *sly* (2), were absent in the YB51 genome. The distribution of virulence-associated genes in the YB51 genome is the same as in the ST3 genome (16).

### Nucleotide sequence accession number. 

The complete genome sequence of *S. suis* serotype 3 (strain YB51) has been assigned GenBank accession no. CP006645.
